# The Difficulties in Emotion Regulation Scale – Short Form (DERS-SF): psychometric properties and invariance between genders

**DOI:** 10.1186/s41155-022-00214-2

**Published:** 2022-05-06

**Authors:** Patrícia Gouveia, Catarina Ramos, José Brito, Telma C. Almeida, Jorge Cardoso

**Affiliations:** 1 Laboratório de Psicologia Egas Moniz (LabPSI), Centro de Investigação Interdisciplinar Egas Moniz (CiiEM), Instituto Universitário Egas Moniz, Campus Universitário, Quinta da Granja, Monte de Caparica, 2829-511 Caparica, Portugal; 2 WDXRFLab, Center for Interdisciplinary Research Egas Moniz (CIIEM), Health Sciences Institute, Monte de Caparica, Portugal

**Keywords:** Emotion regulation, Assessment, DERS-SF, Psychometrics, Invariance

## Abstract

**Background:**

The understanding of how individuals manage their emotional experiences has flourished dramatically over the last decades, including assessing of emotion (dys)regulation. The Difficulties in Emotion Regulation Scale (DERS) is a well-validated and extensively used self-report instrument for emotion regulation problems. Despite the wide use of DERS in both clinical and research settings, its length potentially increases fatigue and frustration in respondents and limits its inclusion in brief research protocols. Consequently, a short-form version of the DERS (DERS-SF) was developed, which requires cross-cultural adaptations and the study of its reliability and validity.

**Objectives:**

In order to address this issue, this study aimed to analyze the factorial structure and psychometric properties of the Portuguese version of DERS-SF and examine the DERS-SF factor structure invariance between men and women.

**Methods:**

The sample comprised 646 participants aged between 18 and 66 years (*M* = 29.93, *SD* = 11.71).

**Results:**

The correlated six-factor structure of the original version has an acceptable fit, good reliability, and convergent validity. Our results also suggested the invariance of the factor structure of the DERS-SF across genders.

**Conclusion:**

The DERS-SF has good psychometric properties, and it may be useful for future research and clinical work to use this six-factor brief version and improve emotion regulation assessment.

## Introduction

Historically, the concepts of emotion and emotional regulation have been widely debated, as well as what characterizes the difficulties in emotional regulation, also known as emotional dysregulation. Emotions can be defined as transient changes in an individual’s subjective experiences, behaviors, and physiological responses, resulting from motivationally relevant internal or external stimuli (Gross, 2015; Lang, 1995). According to the functional perspective, emotions have an adaptive purpose, that is, they are promoters of survival (Kehoe & Havighurst, 2020).

The ability to manage emotions and express them appropriately requires skills in identifying, understanding, and regulating emotions in both intra- and interpersonal situations (Halberstadt et al., 2001). Emotion regulation involves monitoring, evaluating, and modifying emotional reactions (Thompson, 1994). It can be defined as a process that aims to intensify, decrease, or maintain the behavioral, cognitive, experiential, or physiological valences of emotion, depending on the subject’s objectives (Gross & Thompson, 2007). The effectiveness of emotion regulation implies the selection of appropriate strategies and flexibility in their use, which in itself means psychological adjustment (Campbell-Sills & Barlow, 2006). Deficits in emotion regulation are associated with psychopathology symptoms, other emotion-related constructs, and therapy progress (Victor & Klonsky, 2016).

Recognizing the importance of the functional role of emotions, Gratz and Roemer (2004) proposed a multifactorial conceptualization of emotion regulation, formed by the following dimensions: consistency and understanding of emotions; acceptance of emotions; ability, in the face of negative emotions, to control impulsive behaviors and act in line with the desired goals; and ability to use flexible and appropriate emotion regulation strategies, modulating emotional responses to achieve individual goals and deal with the demands of the situation. The relative absence of any or all these capacities may indicate the presence of difficulties in emotion regulation or emotion dysregulation. Usually, emotion regulation skills increase with age (Orgeta, 2009). However, difficulties in regulating emotions can occur throughout the life cycle (Kaufman et al., 2016).

Two different approaches to the concept of emotion regulation supported the development of self-report instruments: (1) emotion dysregulation responses in the form of emotional sensitivity, reactivity, and/or intensity and (2) maladaptive responses to emotions, regardless of the characteristics of the emotions (Gratz et al., 2020). The Difficulties in Emotion Regulation Scale (DERS) developed by Gratz and Roemer (2004) is based on the second perspective, which emphasizes the functional nature of emotions. There are other measures to evaluate this construct (e.g., Catanzaro and Mearns Generalized Expectancy for Negative Mood Regulation Scale; Trait Meta-Mood Scale; Emotional Regulation Questionnaire), but they all assess how individuals’ internal experiences impact their affective response and only reflect a single aspect or subset of emotions (Mekawi et al., 2021). Conversely, the DERS was designed to assess trait-level perceived emotion regulation ability in a multidimensional and comprehensive regulation perspective (Gratz & Roemer, 2004).

The DERS (Gratz & Roemer, 2004) is an extensively used instrument in different populations worldwide, covering adults and adolescents and presenting comprehensive empirical support (Charak et al., 2019). This self-response scale aims to assess clinically significant emotion regulation difficulties through 36 items that fall into 6 domains: non-acceptance of negative emotions (Non-Acceptance), inability to engage in goal-oriented behaviors when experiencing negative emotions (Goals), difficulties in controlling impulsive behaviors when experiencing negative emotions (Impulses), restricted access to emotion regulation strategies perceived as effective (Strategies), lack of emotional awareness (Awareness), and lack of emotional clarity (Clarity).

Numerous studies have confirmed this six-factor structure (Fowler et al., 2014; Gratz & Roemer, 2004; Neumann et al., 2010), although more recently, it has been suggested that items of the Awareness factor could be removed due to their lower validity and consistency (Hallion et al., 2018; Miguel et al., 2017; Osborne et al., 2017).

Contributing to the strength of the convergent validity, it was found that the emotional difficulties measured by this scale are significantly associated with a multiplicity of behaviors, such as self-mutilation, domestic violence, binge eating, substance abuse, and risky behaviors; psychopathological conditions, including borderline personality disorder, post-traumatic stress disorder, anorexia nervosa, social anxiety disorder, and behavioral disorder; and with countless other constructs in the scope of mental functioning, namely negative affect, the severity of depressive and anxious symptoms, tolerance to distress, experiential avoidance, and self-compassion (for a review see Gratz et al., 2020).

Given its relevance, DERS was translated and validated for several countries, originating multiple versions, including a Portuguese-European version (Coutinho et al., 2010) and a Portuguese-Brazilian version (Miguel et al., 2017), both with good psychometric qualities. Despite the wide use of DERS in both clinical and research contexts, its length represents an important limitation, given the expected burden effects on respondents and the difficulties associated with time constraints, particularly in assessments with close intervals and in large epidemiological studies (Kaufman et al., 2016; Shahabi et al., 2020). In addition, due to the similarity of some items, these tend to be perceived as repetitive, increasing fatigue and frustration, which advises the use of short instruments that can be equally effective in evaluating the same construct (Kaufman et al., 2016).

Considering these factors, three short versions of the DERS were developed: the Difficulties in Emotion Regulation Scale – Short Form (DERS-SF) (Kaufman et al., 2016), the DERS-18 (Victor & Klonsky, 2016), and the DERS-16 (Bjureberg et al., 2016). When comparing the DERS short forms (DERS-SF, DERS-18, DERS-16), all the three scales showed strong concordance with the original extended version, internal consistency fair-to-good, and reliability above 0.80 for all subscales except Awareness (Hallion et al., 2018). The authors of this comparative study reported no evidence that any of the short forms were psychometrically superior to the others. However, short versions that retained subscale scores (i.e., DERS-SF and DERS-18) have shown strong concurrent validity, given their capacity to predict current symptoms of anxiety and depression (Skutch et al., 2019). Considering that the short forms generally performed similarly to the original DERS, despite a slight loss of predictive utility (1–3% of the variance) to explain clinical severity, it was suggested that its use is acceptable in most clinical and research situations, with the long form of DERS being indicated for when a comprehensive assessment is required (Hallion et al., 2018; Shahabi et al., 2020). The DERS-SF has been the most used (Skutch et al., 2019) and the most recommended version intended to investigate emotion dysregulation throughout the life cycle, as it is the only scale that presents invariance among adolescents and adults (Charak et al., 2019). The DERS-SF is composed of half of the items in the original version and was developed using two adult and three adolescent samples, verifying the maintenance of the six-factor structure and the excellent psychometric properties of the original instrument, regardless of the age variation.

As with DERS, these short instruments also need to be subject to cross-cultural adaptations and the respective study of their psychometric properties. Recently, a study carried out in Portugal by Moreira et al. (2020), with an adolescent and adult community women sample and aiming to contribute to clarifying the adequacy of the DERS-SF, suggested a bifactorial model without the Awareness subscale.

Pointing out inconsistencies in the literature about the multidimensionality of DERS and its short forms (e.g., Hallion et al., 2018; Osborne et al., 2017), Moreira et al. (2020) tested several models for the scale structure. Between the first group of models tested, a correlated six-factor model and a bifactorial model exhibited a good fit to the data. Nevertheless, the former presented high correlations between all factors except for the correlations between Awareness and all the other factors, and the second had some problems with some items that had non-significant loading or weak factor loading. Considering the results of the first models, they tested a second set of models but removed the Awareness subscale. The correlated six-factor model without Awareness had a good fit to the data, but the bifactorial model without Awareness had a very good and better internal consistency of the subscales. Moreira et al. (2020) suggested that the items on the Awareness subscale should not be included in calculating the total value of the DERS-SF. The authors suggested that the Awareness subscale may be interpreted as a unique measure that evaluates difficulties in processing emotions, and not in the regulation of emotional responses.

Research is moderately extensive but far from conclusive concerning the differences between men and women in terms of emotion regulation and their difficulties. Nolen-Hoeksema and Aldao (2011) found that compared to men, women reported a wider range of emotion regulation strategies, including acceptance, social support, problem-solving reassessment, and rumination, being more likely to resort to more adaptive strategies at older ages. Furthermore, Gratz and Roemer (2004) during the validation of DERS found that men had greater difficulties in becoming emotionally aware. There were differences in the Clarity domain in the DERS validation studies for the Portuguese population, with men showing more difficulties recognizing their emotions (Veloso et al., 2011). However, this finding was not corroborated by a recent study, which found a greater lack of emotional clarity in female participants (Shahabi et al., 2020). In summary, gender differences have been documented in some DERS subscales, albeit sometimes inconsistently. It should be noted that these differences are often not found in the instrument’s overall score (Gratz & Roemer, 2004; Tull et al., 2012). However, this analysis has not yet been carried out using the DERS-SF. At the same time, considering the articulation between gender differences and factorial invariance, and considering that this has never been investigated, it is pertinent to explore gender invariance.

Based on a sample of adults from the general population, the present study aims to (a) analyze the factorial structure and psychometric properties of the Portuguese version of DERS-SF (Kaufman et al., 2016) and (b) analyze the DERS-SF factor structure invariance between men and women.

## Method

### Participants

The sample was composed of 646 subjects, 400 (61.9%) women, and 246 (38.1%) men of Portuguese nationality, aged between 18 and 66 years (*M* = 29.93, *SD* = 11.71). Most participants were single (*n* = 446, 69.0%), employed (*n* = 338, 52.3%), and had completed secondary education (*n* = 291, 45.0%) and higher education (*n* = 321, 49.7%).

### Instruments

#### Sociodemographic questionnaire

A brief questionnaire was developed to assess the following sociodemographic variables: gender, age, marital status, occupational status, and educational level.

#### Difficulties in Emotion Regulation Scale – Short Form (DERS-SF)

The Difficulties in Emotion Regulation Scale – Short Form (DERS-SF; Kaufman et al., 2016) is a scale used to assess the difficulties in emotion regulation. This instrument is based on the Difficulties in Emotion Regulation Scale (Gratz & Roemer, 2004), which is a well-validated and frequently used instrument in assessing this construct. The original scale is composed of 36 items (Gratz & Roemer, 2004), whereas the short version comprises18 items, divided into six subscales, each composed of three items: Strategies, Non-Acceptance, Impulse, Goals, Awareness, and Clarity. The answers are assessed using a 5-point Likert scale, from 1 (almost never) to 5 (almost always). It is possible to obtain the score of each subscale, and the total score of the DERS-SF, with higher values indicating greater difficulties in emotion regulation. The original DERS-SF validation with two adults samples revealed excellent psychometric properties, with the following Cronbach’s alphas: Clarity (0.78), Awareness (0.78), Strategies (0.82), Non-Acceptance (0.85), Impulse (0.89), Goals (0.91), and 0.89 for the total scale (Kaufman et al., 2016).

### Procedure

For the present study, the items of the DERS-SF were translated from the original version (Kaufman et al., 2016) to Portuguese by fluent researchers in both languages and then back-translated from Portuguese to English. The translated version was applied to a small, randomly selected sample of men and women to discuss in detail the understanding of the items and to obtain the final translation of the Portuguese version of the DERS-SF.

Subsequently, the instrument was applied to the general population through an online questionnaire platform (i.e., Google Forms). Participants’ recruitment was carried out by disseminating the study through personal contact networks and social networks. All study participants were clarified about the objectives and procedures of the study and confidentiality and anonymity of the data and gave their informed consent before completing the questionnaire. The study was previously approved by the University Ethics Committee.

### Data analysis

The original sample (*n* = 646) was randomly divided into subsamples S1 (*n* = 327) and S2 (*n* = 319). Exploratory factor analysis (EFA) and confirmatory factor analysis (CFA) were performed in subsamples S1 and S2, respectively.

Exploratory factor analysis (EFA) was performed for simplification of the 18 interrelated measures of the DERS scale, to uncover patterns in that set of variables. Factor extraction was conducted using the principal axis factoring (PAF), and suitable orthogonal (VARIMAX) and oblique (Direct oblimin and Promax) factor rotation techniques were explored. The number of factors to retain was decided upon the results of a parallel analysis (PA) performed using the JASP Computer Software (ASP Team (2021). JASP (version 0.16) [computer software]).

For the following analyses, data were treated using the SPSS statistics software (IBM SPSS Statistics®, v.27.0, IBM® Corp, Armonk, NY) and AMOS® (v.27.0, SPSS Inc., Chicago, IL) for a significance level of *α* ≤ 0.05. Confirmatory factor analyses (CFA), with maximum likelihood as the estimation method, were performed in order to compare the factor structure resulting from the EFA, which was equal to the six-factor structure obtained in the original article (Kaufman et al., 2016), with the bifactorial model of a recent Portuguese version of DERS Short Form (Moreira et al., 2020). The model fit was assessed based on the following indexes: chi-square (*χ*^2^/df < 2), goodness-of-fit index (GFI > 0.90), comparative fit index (CFI > 0.90), and the root mean square of approximation (RMSEA < 0.05) (Marôco, 2014).

For the assessment of the psychometric properties, within the scope of the internal structure of the selected factor structure, reliability was assessed according to Cronbach’s alpha (> 0.70) (Pestana & Gageiro, 2008), and according to the composite reliability (> 0.70), for the subscales. Convergent validity was assessed through average variance extracted (AVE ≥ 0.50), and discriminant validity was calculated by comparing the AVE with the square of the correlations between the factors (AVEi and AVEj ≥ ρij) (Marôco, 2014).

We proceeded with the multi-group analysis using the stepwise procedure to assess the factor invariance of the DERS-SF across genders, with S2. Four models that differed from each other according to the following sets of parameters were used to answer questions related to multi-group equivalence: model 2a—unconstrained; model 2b—factor loadings; model 2c—structural covariances; and model 2d—measurement residuals (Byrne, 2010; Marôco, 2014). Invariance was calculated by comparing the difference of the CFI (ΔCFI) regarding the baseline model (i.e., unconstrained; model 2a) and resulting models (fixed factor loadings, structural covariances, and measurement residuals; models 2b–2d). Thus, gender invariance was verified when the values of ΔCFI were less than 0.01 (Byrne, 2010). According to Cheung & Rensvold (2002) cit in Byrne (2010), “it may be more reasonable to base invariance decisions on a difference in CFI (ΔCFI) rather than on *χ*^2^ values” (p. 221).

Finally, descriptive analysis of the DERS-SF was calculated for the subsamples of men and women, in parallel with the comparison of means between these groups, using one-way analysis of variance (ANOVA). The one-way ANOVA’s assumptions were previously verified.

## Results

### Evidence of validity based on the internal structure

#### Factor analysis

To begin, according to PA, 6 factors must be retained in this analysis, based on the comparison of the actual eigenvalues and the 95th percentile of the eigenvalues derived from the random data matrices with the actual size (327 cases, 18 variables).

Moreover, EFA using Varimax rotation has resulted in a factor transformation matrix which off-diagonal elements deviate markedly from a near symmetry (data not shown) suggesting that the DERS factors may be correlated, and thus, oblique rotation may support a more realistic theoretically explanation.

In these conditions, the Direct oblimin and Promax rotation techniques were applied which produced pattern matrices that differ only slightly in the saturation of one item (item 10) in different factors. Thus, EFA suggested one model which was further explored in CFA. This model had the same factorial structure as the model in the original article by Kaufman et al. (2016) (Table 1). Moreover, the resulting correlation matrix between factors shown in Table 2 for EFA with Oblimin rotation presents several correlations in excess of 0.32, which is indicative of variance overlapping between factors, thus warranting the applied oblique rotation.

**Table 1:** Pattern matrix: factor loadings < 0.35 suppressed

	Factor^a^					
	1	2	3	4	5	6
Item 1			0.713			
Item 2				0.500		
Item 3				0.840		
Item 4			0.832			
Item 5				0.606		
Item 6			0.544			
Item 7					0.718	
Item 8	0.728					
Item 9		0.611				
Item 10						0.379
Item 11	0.870					
Item 12					0.685	
Item 13	0.864					
Item 14		0.781				
Item 15						0.645
Item 16					0.357	
Item 17		0.864				
Item 18						0.542

^a^Extraction method: principal axis factoring; rotation method: Oblimin without Kaiser normalization

**Table 2:** Correlation matrix for the 6 factors in the EFA with direct Oblimin rotation for the DERS data

Factor^a^	2	3	4	5	6
1	0.672	− 0.186	0.518	0.634	0.644
2	–	0.039	0.493	0.589	0.552
3		–	0.138	− 0.039	0.021
4			–	0.573	0.591
5				–	0.623
6					–

^a^Extraction method: principal axis factoring; rotation method: Oblimin without Kaiser normalization

**Fig. 1:**
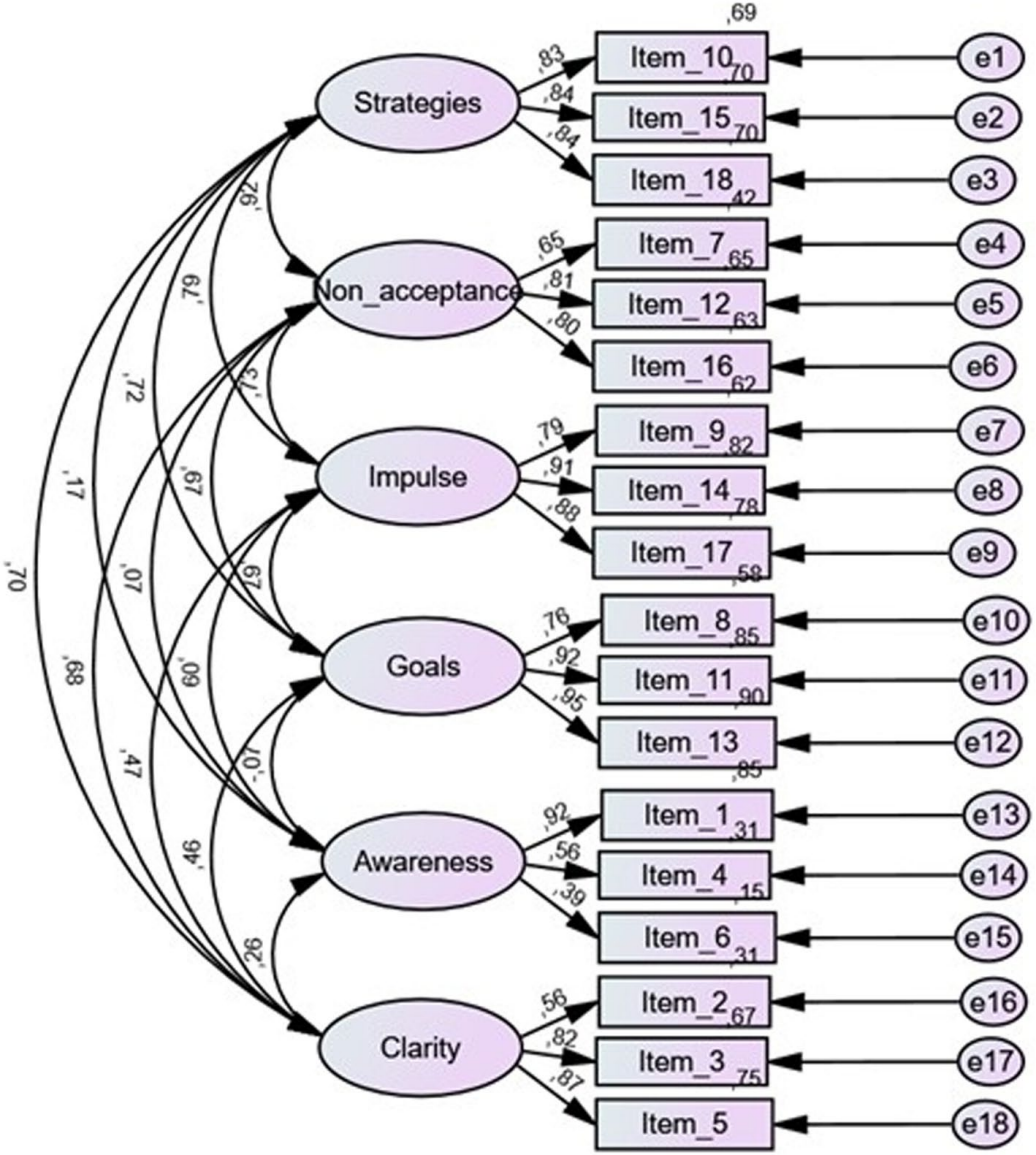
Correlated six-factor structure of the DERS-SF

A confirmatory factor analysis was conducted to assess which factorial structure had a better model fit among the following models: model 1 bifactor model excluding the Awareness subscale (Moreira et al., 2020) and model 2—correlated six-factor model resulting from EFA and original article (Kaufman et al., 2016). Table 3 displays the model fit indexes of the different factor structures (models 1–2). The correlated six-factor structure as stated in the EFA and the original validation article (Kaufman et al., 2016) presents a good model fit for the present sample (*χ*^2^ (120) = 245,309; *p* ≤ 0.001; GFI = 0.921; CFI = 0.965; RMSEA = 0.057 [0.047; 0.068]). Comparing models 1 and 2, despite the first has a higher CFI and GFI than model 1, we select model 2, since in our sample the Awareness subscale did not show any limitations that would justify its exclusion, namely in terms of factor loadings or significance (Fig. 1, Tables 3 and 4).

**Table 3:** Model fit indexes of two factor structures of DERS-SF^a^ and factorial invariance for gender (*n* = 319)

Model	*χ*^2^	df	*χ*^2^/df	GFI	CFI	RMSEA	C.I.90%
1. Bifactor model without awareness (Moreira et al., 2020)	139.308*	65	2.143	0.944	0.978	0.060	0.046; 0.074
2. Correlated six-factor structure^b^	245.309*	120	2.044	0.921	0.965	0.057	0.047; 0.068
2a. Unconstrained	387.387*	240	1.614	0.884	0.958	0.044	0.036; 0.052
2b. Factor loadings	395.036*	252	1.568	0.882	0.960	0.042	0.034; 0.050
2c. Structural covariances	414.078*	273	1.517	0.878	0.960	0.040	0.032; 0.048
2d. Measurement residuals	428.470*	291	1.472	0.874	0.961	0.039	0.031; 0.046

* *p* ≤ 0.001

^a^*DERS-SF* Difficulties in Emotion Regulation Scale – Short Form

^b^This model results from the similar factorial structure obtained in EFA and by Kaufman et al. (2016)

**Table 4:** Factor loadings of correlated six-factor structure

Items	Factor	*λ*
1. Presto atenção a como me sinto. [I pay attention to how I feel.]	Awareness	0.92
2. Não tenho ideia de como me sinto. [I have no idea how I am feeling.]	Clarity	0.56
3. Tenho dificuldade em entender os meus sentimentos. [I have difficulty making sense out of my feelings.]	Clarity	0.82
4. Preocupo-me com o que sinto. [I care about what I am feelings.]	Awareness	0.56
5. Estou confuso/a com o que sinto. [I am confused about how I feel.]	Clarity	0.87
6. Quando estou transtornado/a reconheço as minhas emoções. [When I’m upset, I acknowledge my emotions.]	Awareness	0.39
7. Quando estou transtornado/a fico envergonhado/a por me sentir assim. [When I’m upset, I become embarrassed for feeling that way.]	Non-Acceptance	0.65
8. Quando estou transtornado/a tenho dificuldade em concluir tarefas. [When I’m upset, I have difficulty getting work done.]	Goals	0.76
9. Quando estou transtornado/a fico descontrolado/a. [When I’m upset, I become out of control.]	Impulse	0.79
10. Quando estou transtornado/a acredito que vou acabar por me sentir muito deprimido/a. [When I’m upset, I believe that I will end up feeling very depressed.]	Strategies	0.83
11. Quando estou transtornado tenho dificuldade em focar-me noutras coisas. [When I’m upset, I have difficulty focusing on other things.]	Goals	0.92
12. Quando estou transtornado/a sinto-me culpado/a por me sentir assim. [When I’m upset, I feel guilty for feeling that way.]	Non-Acceptance	0.81
13. Quando estou transtornado/a tenho dificuldade em concentrar-me. [When I’m upset, I have difficulty concentrating.]	Goals	0.95
14. Quando estou transtornado/a tenho dificuldade em controlar os meus comportamentos. [When I’m upset, I have difficulty controlling my behavior.]	Impulse	0.91
15. Quando estou transtornado/a acredito que não há nada que eu possa fazer para me sentir melhor. [When I’m upset, I believe there is nothing I can do to make myself feel better.]	Strategies	0.84
16. Quando estou transtornado/a. fico irritado/a comigo por me sentir assim. [When I’m upset, I become irritated at myself for feeling that way.]	Non-Acceptance	0.80
17. Quando estou transtornado/a. perco o controlo sobre o meu comportamento. [When I’m upset, I lose control over my behavior.]	Impulse	0.88
18. Quando estou transtornado/a. demoro muito tempo a sentir-me melhor. [When I’m upset, it takes me a long time to feel better.]	Strategies	0.84

##### Reliability, convergent, and discriminant evidence

The analysis of reliability was performed by assessing Cronbach’s alpha, whose value should be higher than 0.70 (Pestana & Gageiro, 2008). The values found in the Portuguese version of the DERS-SF presented a Cronbach’s alpha of the following: Awareness: *α* = 0.70; Clarity: *α* = 0.77; Non-Acceptance: *α* = 0.78; Strategies: *α* = 0.86; Impulse: *α* = 0.88; and Goals: *α* = 0.91 and *α* = 0.90 for the total scale. Since the factor structure of the DERS-SF has 6 factors, composite reliability (CR) was also performed, and all subscales had scores ranging between 0.67 (Awareness) and 0.91 (Goals). As regards convergent validity, all subscales were confirmed to have an average variance extracted (AVE) higher than 0.50, except for the subscale Awareness (AVE = 0.44). The remaining subscales have AVE values between 0.57 (Non-Acceptance) and 0.78 (Goals). Regarding discriminant validity, we confirmed its existence between all subscales since the AVE of each subscale is higher than the square of the correlation between each pair of subscales. The subscales Strategies and Non-Acceptance showed problems concerning discriminant validity, since the square of the correlation 0.92 is higher than the AVE of the subscale Strategies (AVE = 0.70) and Non-Acceptance (AVE = 0.57).

##### Invariance of the factor structure

Considering the statistically significant differences between genders, we conducted the multi-group CFA analysis to assess the factor invariance between genders by comparing the four models (model 2a to model 2d—Table 3). The factor invariance analyses between unconstrained model (i.e., model 2a) and resulting models (fixed factor loadings, structural covariances, and measurement residuals; models 2b–2d) revealed a ΔCFI < 0.01, which indicate the invariance of the factor structure of the DERS-SF across genders for the factor loadings, covariances, and residuals.

#### Descriptive analysis and gender differences

The descriptive analysis of the DERS-SF for the total sample can be seen in Table 5 and for the genders can be seen in Table 6. The mean (standard deviation) for the total DERS-SF sample was 38.39 (12.49). The mean for the total DERS-SF was 37.72 (12.13) in men and 39.13 (12.69) in women. Statistically significant differences were identified only between these two groups for the Strategies subscale (*F*(1, 644) = 4.70, *p* = 0.031).

**Table 5:** Descriptive analysis for total sample (*N* = 646)

	*M*	*SD*	*Sk*	*Ku*
Strategies	6.12	3.06	1.11	0.52
Goals	7.79	3.39	0.49	-0.71
Impulse	5.53	2.86	1.30	1.15
Clarity	5.77	2.56	1.20	1.33
Awareness	7.05	2.82	0.61	-0.16
Non-Acceptance	6.34	2.86	0.86	0.16
Difficulties in emotion regulation—total score	38.59	12.49	0.79	0.08

*M* mean, *SD* standard deviation, *Sk* skewness, *Ku* kurtosis

**Table 6:** Descriptive analysis and gender differences of DERS-SF

Variable	Men sample (*n* = 246)			Women sample (*n* = 400)			*F* (1, 644)^a^
	*α*	*M*	*SD*	α	*M*	*SD*	
Strategies	0.87	5.78	2.95	0.85	6.32	3.11	4.699*
Goals	0.92	7.48	3.45	0.90	7.98	3.34	0.874
Impulse	0.89	5.42	2.79	0.87	5.60	2.91	0.552
Clarity	0.76	5.63	2.47	0.78	5.86	2.61	3.456
Awareness	0.73	7.20	2.89	0.69	6.95	2.78	1.168
Non-Acceptance	0.75	6.20	2.66	0.79	6.42	2.98	1.230
Difficulties in emotion regulation total score	0.90	37.72	12.13	0.90	39.13	12.69	1.965

*α* Cronbach’s alpha, *M* mean, *SD* standard deviation, *DERS-SF* Difficulties in Emotion Regulation Scale – Short Form

*p < 0.05

^a^One-way ANOVA

## Discussion

In this study, psychometric properties, factor structure, and gender invariance of the DERS-SF were analyzed in a sample of the Portuguese population.

Inconsistencies remain in the literature regarding the most appropriate structure of DERS (Victor & Klonsky, 2016) and which versions of DERS are best suited to different contexts (e.g., clinical, research). To clarify the factorial structure of the short form of DERS, other studies compared the different versions of the scale (i.e., DERS-16, DERS-SF, and DERS-18), in a sample of undergraduate students (Skutch et al., 2019); among adults and adolescents with severe mental illness (Charak et al., 2019); and with adults with one or more emotional disorders according to the DSM-5 (Hallion et al., 2018). The results of Skutch et al. (2019) revealed that the reliability and validity of the three structures are identical, but despite that, the authors recommended using the DERS-SF or DERS-18 version, as they allow the addition of sums for the subscales. In our study, we ascertained the relevance of DERS-SF, but only with a normative sample.

In order to confirm the factor structure of DERS-SF, two different factorial structures of the DERS-SF were compared: (a) bifactorial model excluding the Awareness subscale recently studied in a Portuguese sample (Moreira et al., 2020); and (b) correlated six-factor structure resulting from exploratory factor analysis with study sample, equal to factor structure of correlated six factors resulting from the original DERS-SF (Kaufman et al., 2016), and (b) bifactorial model excluding the Awareness subscale recently studied in a Portuguese sample (Moreira et al., 2020). The results obtained demonstrated that the factor structure of the correlated six factors resulting from the EFA, equal to the original version with a sample of adolescents and university students (Kaufman et al., 2016), is the one that presents a better model fit to the sample (including all the subscales) of the present study, which is following other studies (Charak et al., 2019; Skutch et al., 2019). The mean age in our sample represents young adults, which may justify that the same factor structure also demonstrated a good adjustment in our study. Moreira et al. (2020) used a sample of female adults and adolescents from the Portuguese normative population. Additionally, the 18 items used in this study were extracted from the Portuguese version of 36 items (Coutinho et al., 2010) and were not the result of the translation process of the original version of the DERS-SF (Kaufman et al., 2016), as in our study. In this sense, differences in the characteristics of our and Moreira’s et al. (2020) sample, and the sentence content of the items, may justify the disparity in the results. Unlike Moreira et al. (2020) study, in our correlated six-factor model, the Awareness subscale did not present factor loadings, reliability, or correlations with the total scale or subscales that justified its exclusion. We consider that validation studies of DERS-SF with a representative sample of the Portuguese population are still recommended.

In this sample of the Portuguese population, DERS-SF has good psychometric properties, in particular, high factorial validity, similar to the results of other studies (Charak et al., 2019; Kaufman et al., 2016). Reliability is also close to the results found in the original study (Kaufman et al., 2016) as well in the study conducted by Skutch et al. (2019), although in this one, the subscales Clarity, Non-Acceptance, and Awareness have higher values of reliability when compared to our study’s results. Regarding discriminant validity, the Awareness subscale showed good correlations with the remaining subscales, contrary to what was found in other studies of the DERS-SF version (Moreira et al., 2020) or of the DERS-18 version (Victor & Klonsky, 2016). However, in the present study, the Awareness subscale was not problematic in terms of conceptual discrimination with the remaining subscales, and problems with discriminant validity were found only between the Strategies and Non-Acceptance subscales.

Concerning gender invariance, the differences in the difficulties in namely in Strategies subscale, between men and women were found in our sample, and other studies (e.g., Gratz & Roemer, 2004; Nolen-Hoeksema & Aldao, 2011; Veloso et al., 2011) supported the analysis of the DERS-SF factorial invariance between genders. The DERS-SF factor structure invariance between genders was verified, for factor loadings, covariance, and residuals. This result suggests that assessing the difficulties in emotion regulation can be carried out using the same factorial structure of DERS-SF for men and women since both groups have the same understanding of the constituent items of latent scale dimensions.

The results of the present study must be interpreted by taking into account the following limitations. First, the non-probabilistic and convenience sampling, the higher percentage of women, and the low average age of the participants prevent generalizing the results to the Portuguese population. Future studies are recommended with greater representativeness in terms of age and gender. Second, the participants are from the non-clinical population, and variables related to maladaptive circumstances, including psychological distress or mental disorder, were not controlled. Third, the non-performance of test-retest and the concurrent validity of DERS-SF, compared to DERS-36 (Kaufman et al., 2016; Skutch et al., 2019), is also an important limitation. Finally, the factorial invariance was tested for gender only, and therefore, no other conclusions can be drawn regarding invariance between the groups defined according to other socio-demographic or clinical characteristics.

Despite the limitations mentioned above, the present study contributes to the cross-cultural dissemination of this instrument, namely, among Portuguese speakers.

## Data Availability

The datasets generated during and/or analyzed during the current study are available from the corresponding author on reasonable request.

## Abbreviations

DERS-SF: Difficulties in Emotion Regulation Scale – Short Form
EFA: Exploratory factor analysis
PAF: Principal axis factoring
PA: Parallel analysis
KMO: Kaiser-Meyer-Olkin indicator
*χ*^2^: Chi-square
df: Degrees of freedom
CFA: Confirmatory factor analysis
GFI: Goodness-of-fit index
CFI: Comparative fit index
RMSEA: Root mean square error of approximation
AVE: Average variance extracted
CR: Composite reliability
ANOVA: Analysis of variance
M: Mean
SD: Standard deviation
Sk: Skewness
Ku: Kurtosis
