# Correction: The Difficulties in Emotion Regulation Scale – Short Form (DERS-SF): psychometric properties and invariance between genders

**DOI:** 10.1186/s41155-022-00225-z

**Published:** 2022-07-04

**Authors:** Patrícia Gouveia, Catarina Ramos, José Brito, Telma C. Almeida, Jorge Cardoso

**Affiliations:** 1 Laboratório de Psicologia Egas Moniz (LabPSI), Centro de Investigação Interdisciplinar Egas Moniz (CiiEM), Instituto Universitário Egas Moniz, Campus Universitário, Quinta da Granja, Monte de Caparica, 2829-511 Caparica, Portugal; 2 WDXRFLab, Center for Interdisciplinary Research Egas Moniz (CIIEM), Health Sciences Institute, Monte de Caparica, Portugal

**Psicologia: Reflexão e Crítica (2022) 35:1-10**

**https://doi.org/10.1186/s41155-022-00214-2**

Following publication of the original article (Gouveia et al., 2022), the below funding note was missing.

Funding

This work is financed by national funds the FCT - Foundation for Science and Technology, I.P., under the project UIDB/04585/2020.

The original article (Gouveia et al., 2022) has been corrected.
